# Primary lung cancer treatable with radical resection after complete remission with pembrolizumab therapy following gemcitabine and carboplatin chemotherapy for multiple metastases of bladder cancer

**DOI:** 10.1002/iju5.12550

**Published:** 2022-11-11

**Authors:** Ryohta Nakamura, Go Hasegawa, Kazumasa Ohashi, Takehisa Hashimoto, Yohei Ikeda, Noboru Hara, Tsutomu Nishiyama

**Affiliations:** ^1^ Department of Urology, Uonuma Institute of Community Medicine Niigata University Medical and Dental Hospital Minamiuonuma Niigata Japan; ^2^ Department of Pathology, Uonuma Institute of Community Medicine Niigata University Medical and Dental Hospital Minamiuonuma Niigata Japan; ^3^ Department of Respiratory Medicine, Uonuma Institute of Community Medicine Niigata University Medical and Dental Hospital Minamiuonuma Niigata Japan; ^4^ Department of Respiratory Surgery, Uonuma Institute of Community Medicine Niigata University Medical and Dental Hospital Minamiuonuma Niigata Japan; ^5^ Department of Diagnostic Radiology, Uonuma Institute of Community Medicine Niigata University Medical and Dental Hospital Minamiuonuma Niigata Japan

**Keywords:** advanced bladder cancer, pembrolizumab therapy following gemcitabine and carboplatin chemotherapy with complete remission, primary lung cancer with radical resection

## Abstract

**Introduction:**

We report a patient with the complete remission of multiple metastases and primary bladder lesions of bladder cancer who developed primary lung cancer requiring radical resection.

**Case presentation:**

A 68‐year‐old man diagnosed with invasive bladder cancer, right hydroureteronephrosis, and multiple metastases were administered six courses of gemcitabine and carboplatin chemotherapy and thereafter has been receiving pembrolizumab therapy. Bladder cancer and multiple metastases decreased in size, whereas a ground‐glass opacity lesion in the lung gradually increased in size. Fluorodeoxyglucose‐positron emission tomography revealed the accumulation of fluorodeoxyglucose in the ground‐glass opacity lesion only. The patient was diagnosed with primary lung cancer and underwent a thoracoscopic lobectomy. Histopathological findings showed ALK‐negative, EGFR L858R mutation‐positive invasive adenocarcinoma with a programmed death‐ligand 1 tumor proportion score of less than 1%.

**Conclusion:**

This is the first case report of patients with the complete remission of multiple metastases of bladder cancer who developed primary lung cancer requiring radical resection.

**Keynote message**

We herein present a case of primary lung cancer that required radical resection after the complete remission of multiple metastases of bladder cancer with immune checkpoint inhibitors following platinum‐based chemotherapy. With advances in treatment methods, the number of patients with late‐stage double primary cancer achieving a cure will increase. Even for a remission case after multidisciplinary therapies, detectable new regions should be managed carefully because of the possibility of primary cancer development.

**Abbreviations & Acronyms**
CT: computed tomography
GGO: ground‐glass opacity
ICIs: immune checkpoint inhibitors
MIBC: muscle‐invasive bladder cancer
PD‐L1: programmed death‐ligand 1

## Introduction

Potential treatments for metastatic urothelial carcinoma include the incorporation of ICIs following platinum‐based chemotherapy and targeted agents.^1^, ^2^ Lung cancer is the leading cause of cancer‐related death and the third most diagnosed cancer in Japan.^3^

We herein present a patient with primary lung cancer that required radical resection after the complete remission of multiple metastases and primary bladder lesions of bladder cancer with ICIs following platinum‐based chemotherapy.

## Case presentation

**Fig. 1:**
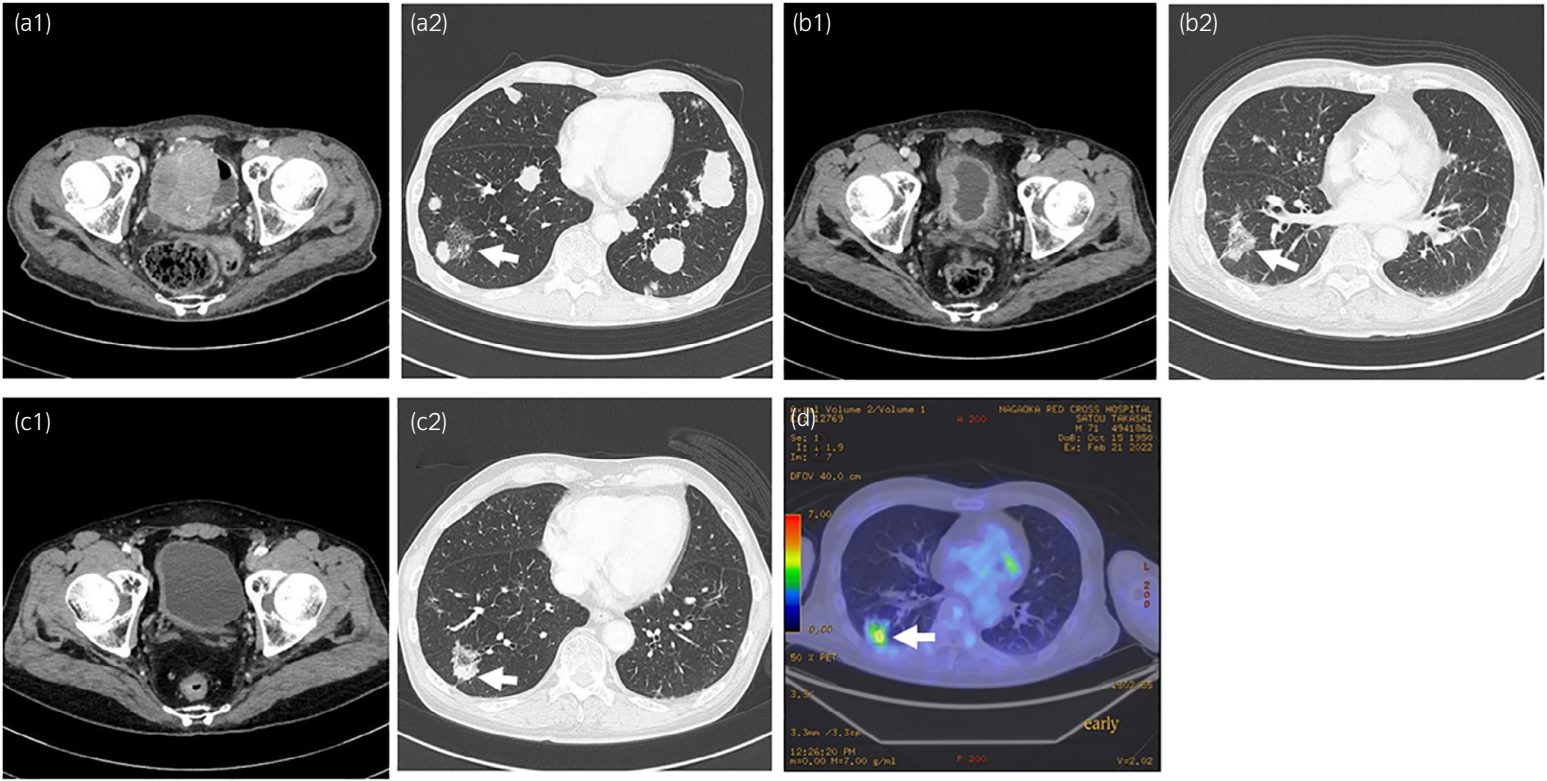
(a) CT at the time of admission showed large lesions in the bladder (a1), multiple lung metastases, and GGO lesion in the inferior lobe of the right lung (a2) (arrow). (b) CT after six courses of gemcitabine and carboplatin chemotherapy revealed decreases in the sizes of bladder cancer (b1) and multiple metastases (b2); however, the GGO lesion was observed in the lower lobe of the right lung (arrow). (c) CT in January 2022 revealed that decreases in the sizes of bladder cancer (c1) and multiple metastases (c2) were maintained during pembrolizumab therapy; however, the GGO lesion in the inferior lobe of the right lung gradually increased in size (arrow). (d) Fluorodeoxyglucose‐positron emission tomography showed the accumulation of fluorodeoxyglucose in the GGO lesion in the inferior lobe of the right lung (arrow).

**Fig. 2:**
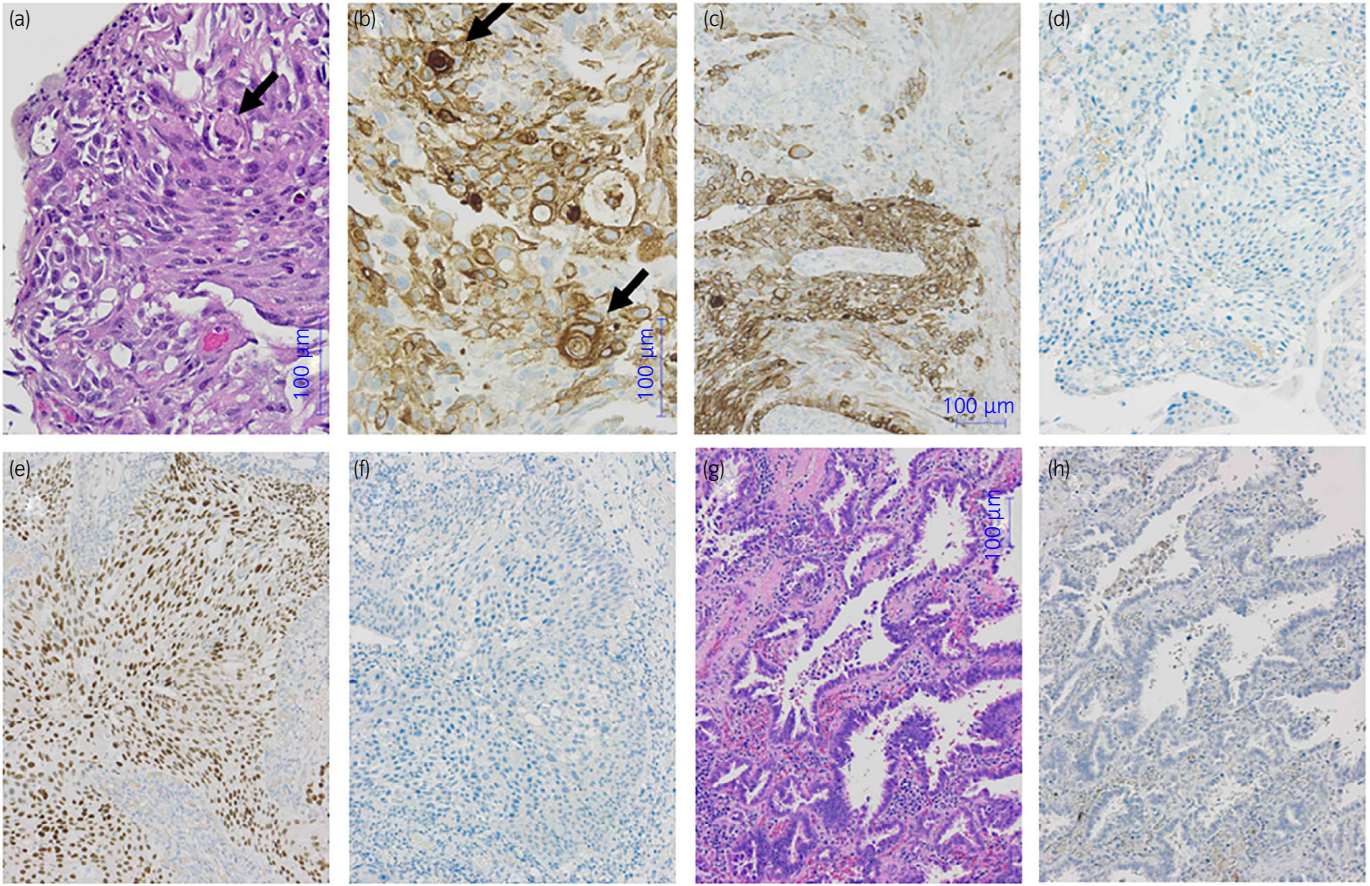
Pathological findings. The pathological findings of bladder cancer showed high‐grade, G2 invasive urothelial carcinoma with squamous differentiation and cancer pearl‐like findings (arrow) (a) hematoxylin–eosin staining, (b) positive for CK5/6 (c), negative for CK20 (d), positive for GATA3 (e), and negative for PD‐L1 (f). The pathological findings of lung cancer showed invasive papillary adenocarcinoma (g: hematoxylin–eosin staining) that was negative for PD‐L1 (h).

A 68‐year‐old man presented with appetite loss, a 25% reduction in body weight, and general fatigue for 8 months in August 2020. Bladder cancer with multiple lung metastases was suspected. Cystoscopy revealed large lesions in the bladder. CT showed large lesions in the bladder with right hydroureteronephrosis, multiple lung metastases, and multiple hilar and mediastinal lymph node metastases, T4aN3M1b (Fig. 1a). The patient underwent transurethral resection for bladder tumors in August 2020. Pathological findings revealed squamous differentiation‐dominant, high‐grade, and G2‐invasive urothelial carcinoma (Fig. 2). Since the patient was ineligible for cisplatin due to his general condition, he received six courses of gemcitabine and carboplatin chemotherapy. Bladder cancer and multiple metastases markedly decreased in size on CT in February 2021 (Fig. 1b). He has been receiving pembrolizumab therapy since February 2021 (200 mg once every 3 weeks, 400 mg once every 6 weeks from October 2021). Cystoscopy showed no protruded lesion and urine cytology revealed negative for urothelial carcinoma in October 2021. Reductions in the size of bladder cancer and multiple metastases were maintained. However, a GGO lesion was detected in the inferior lobe of the right lung and was gradually increasing in size; therefore, primary lung cancer was suspected (Fig. 1c). Bronchoscopy and biopsy were performed but could not obtain a specimen for diagnosis. Fluorodeoxyglucose positron emission tomography revealed the accumulation of fluorodeoxyglucose in the GGO lesion in the inferior lobe of the right lung, but not in the bladder lesion or metastatic sites (Fig. 1d). Based on these findings, a diagnosis of primary lung cancer was reached and the patient underwent a thoracoscopic right lower lobectomy in April 2022. Histopathological findings revealed ALK‐negative, EGFR L858R mutation‐positive invasive adenocarcinoma with a PD‐L1 tumor proportion score of less than 1% (Fig. 2). The patient has been receiving pembrolizumab therapy (400 mg once at 6‐week intervals) without recurrence as of September 2022.

## Discussion

Late‐stage urothelial cancer generally remains an incurable condition. Systemic therapy is important for treatment.^1^ The systemic management of muscle‐invasive (MIBC) and late‐stage bladder cancer has primarily involved platinum‐based chemotherapy. The most commonly used regimens are gemcitabine plus cisplatin and methotrexate, vinblastine, doxorubicin, and cisplatin. Gemcitabine plus carboplatin, a less effective alternative to gemcitabine plus cisplatin for metastatic urothelial cancer, has been examined in retrospective studies on patients with MIBC.^4^

The use of ICIs has improved the survival outcomes of patients with metastatic urothelial cancer.^1^, ^2^ Pembrolizumab achieved significantly longer overall survival (approximately 3 months) than chemotherapy in patients with previously treated metastatic urothelial cancer.^2^

The incidence of bladder cancer metastatic to the lungs is higher in patients with MIBC than in those with non‐MIBC; however, a similar incidence of synchronous second primary lung cancers that was independent of the initial stage of bladder cancer has been reported.^5^ Among patients with MIBC, 0.7% have been diagnosed with synchronous second primary lung cancers. The risk of synchronous lung cancers was found to be 4.7‐fold higher in patients with MIBC than in the general population.

In the present case, decreases in the sizes of invasive bladder cancer and multiple metastases were maintained with pembrolizumab therapy following gemcitabine and carboplatin chemotherapy. The patient was diagnosed with primary lung cancer and underwent thoracoscopic right lower lobectomy. Histopathological findings revealed ALK‐negative, EGFR L858R mutation‐positive invasive adenocarcinoma with a PD‐L1 tumor proportion score of less than 1%. Therefore, pembrolizumab therapy did not appear to be effective against primary lung cancer. In a subset of patients with non‐small cell lung cancer, ICIs were found to markedly prolong survival and improve quality of life. In contrast, patients with EGFR‐mutant non‐small cell lung cancer did not respond well to anti‐PD‐1/PD‐L1 immunotherapy^6^; however, the underlying reasons for poor responses currently remain unclear.

In KEYNOTE‐045, pembrolizumab achieved significantly longer overall survival regardless of the tumor PD‐L1 expression status; however, response rates were higher in patients with tumors expressing elevated levels of PD‐L1.^2^ In the PURE‐01 study, the administration of neoadjuvant pembrolizumab prior to radical cystectomy resulted in the disappearance of residual tumors in 42% of MIBC patients without severe adverse events.^7^ These findings indicate the potential of pembrolizumab as neoadjuvant therapy for MIBC limited to PD‐L1‐positive tumors or those with a high tumor mutational burden. Although bladder cancer tissue in the present case did not express PD‐L1, complete remission was achieved with pembrolizumab therapy following gemcitabine and carboplatin chemotherapy; however, the reasons for this outcome in the present case remain unclear. In KEYNOTE‐045, response rates were higher in patients with tumors with mixed histology than in those with pure urothelial carcinoma.^2^ Pathological findings in the present case revealed urothelial carcinoma with squamous differentiation.

If the patient's current condition can be maintained, we plan to maintain pembrolizumab therapy without radical cystectomy for bladder cancer and follow‐up without additional treatment for lung cancer.

## Conclusion

We encountered a case of primary lung cancer that required radical resection after the complete remission of multiple metastases and primary bladder lesions of bladder cancer with ICIs following platinum‐based chemotherapy. With advances in treatment methods, the number of patients with late‐stage double primary cancer achieving a cure will increase.

## Author contributions

Ryohta Nakamura: Writing – original draft. Go Hasegawa: Writing – review and editing. Kazumasa Ohashi: Writing – review and editing. Takehisa Hashimoto: Writing – review and editing. Yohei Ikeda: Writing – review and editing. Noboru Hara: Writing – original draft. Tsutomu Nishiyama: Writing – original draft; writing – review and editing.

## Conflict of interest

The authors declare no conflict of interest.

## Approval of the research protocol by an Institutional Reviewer Board

Not applicable.

## Informed consent

Written informed consent was obtained from the patient for the publication of this case report and accompanying images.

## Registry and the registration no. of the study/trial

Not applicable.
